# Soil surveillance for monitoring soil-transmitted helminths: Method development and field testing in three countries

**DOI:** 10.1371/journal.pntd.0012416

**Published:** 2024-09-06

**Authors:** Malathi Manuel, Heather K. Amato, Nils Pilotte, Benard Chieng, Sylvie B. Araka, Joël Edoux Eric Siko, Michael Harris, Maya L. Nadimpalli, Venkateshprabhu Janagaraj, Parfait Houngbegnon, Rajeshkumar Rajendiran, Joel Thamburaj, Saravanakumar Puthupalayam Kaliappan, Allison R. Sirois, Gretchen Walch, William E. Oswald, Kristjana H. Asbjornsdottir, Sean R. Galagan, Judd L. Walson, Steven A. Williams, Adrian J. F. Luty, Sammy M. Njenga, Moudachirou Ibikounlé, Sitara S. R. Ajjampur, Amy J. Pickering

**Affiliations:** 1 The Wellcome Trust Research Laboratory, Division of Gastrointestinal Sciences, Christian Medical College Vellore, Vellore, Tamil Nadu, India; 2 Department of Civil and Environmental Engineering, University of California, Berkeley, California, United States of America; 3 Department of Biological Sciences, Quinnipiac University, Hamden, Connecticut, United States of America; 4 Kenya Medical Research Institute, Nairobi, Kenya; 5 Institut de Recherche Clinique du Bénin, Abomey-Calavi, Bénin; 6 Centre de Recherche pour la lutte contre les Maladies Infectieuses Tropicales (CReMIT/TIDRC), Université d’Abomey-Calavi, Calavi, Bénin; 7 Gangarosa Department of Environmental Health, Rollins School of Public Health, Emory University, Atlanta, Georgia, United States of America; 8 Smith College, Northampton, Massachusetts, United States of America; 9 Department of Disease Control, Faculty of Infectious & Tropical Diseases, London School of Hygiene & Tropical Medicine, London, United Kingdom; 10 Global Health Division, International Development Group, RTI International, Research Triangle Park, Durham, North Carolina, United States of America; 11 Center of Public Health Sciences, University of Iceland, Reykjavík, Iceland; 12 DeWorm3, Department of Global Health, University of Washington, Seattle, Washington, United States of America; 13 DeWorm3, Departments of Global Health, Medicine, Pediatrics and Epidemiology, University of Washington, Seattle, Washington, United States of America; 14 Departments of International Health, Medicine and Pediatrics, Johns Hopkins University, Baltimore, Maryland, United States of America; 15 Department of Biological Sciences, Smith College, Northampton, Massachusetts, United States of America; 16 Molecular and Cellular Biology Program, University of Massachusetts Amherst, Amherst, Massachusetts, United States of America; 17 Université Paris Cité, IRD, MERIT, Paris, France; 18 Blum Center for Developing Economies, University of California, Berkeley, California, United States of America; 19 Chan Zuckerberg Biohub, San Francisco, California, United States of America; University of Sussex, UNITED KINGDOM OF GREAT BRITAIN AND NORTHERN IRELAND

## Abstract

**Background:**

One-fifth of the global population is infected with soil-transmitted helminths (STH). Mass drug administration (MDA) with deworming medication is widely implemented to control morbidity associated with STH infections. However, surveillance of human infection prevalence by collecting individual stool samples is time-consuming, costly, often stigmatized, and logistically challenging. Current methods of STH detection are poorly sensitive, particularly in low-intensity and low-prevalence populations.

**Methodology/Principal findings:**

We aimed to develop a sensitive and specific molecular method for detecting STH DNA in large volumes of soil (20 g) by conducting laboratory and proof of concept studies across field sites in Kenya, Benin, and India. We collected human stool (n = 669) and soil (n = 478) from 322 households across the three study sites. We developed protocols for DNA extraction from 20 g of soil and qPCR to detect *Ascaris lumbricoides*, *Trichuris trichiura*, *Necator americanus*, and *Ancylostoma duodenale*. Agreement between detection of STH via qPCR, digital droplet PCR (ddPCR), and microscopy-based methods was assessed using the Cohen’s Kappa statistic. Finally, we estimated associations between soil characteristics and detection of STH in soil by qPCR, as well as between STH detected in soil and STH detected in stool from matched households, adjusting for soil characteristics. The overall prevalence of STH in soil by qPCR was 31% for *A*. *lumbricoides*, 3% for *T*. *trichiura*, and 13% for any hookworm species. ddPCR and qPCR performed similarly. However, there was poor agreement between STH detected in soil by qPCR versus light microscopy. Microscopy underestimated the prevalence of *A*. *lumbricoides* and *N*. *americanus* and overestimated *T*. *trichiura*. Detection of an STH species in household soil was strongly associated with increased odds of a household member being infected with that same species.

**Conclusions/Significance:**

Soil surveillance for STH has several benefits over stool-based surveillance, including lower cost and higher success rates for sample collection. Considering that delivery of MDA occurs at the community level, environmental surveillance using molecular methods could be a cost-effective alternate strategy for monitoring STH in these populations.

## Author summary

Soil surveillance to determine soil-transmitted helminth (STH) prevalence in communities with MDA programs could be cost-effective. Though microscopic methods for the detection of STH in soil exist, they are laborious. With recent increased availability of DNA extraction kits and polymerase chain reaction (PCR) reagents, using molecular assays are promising and provide the ability to discriminate between closely related helminth species. In this study, we developed a sensitive and specific molecular method for the detection of STH in large quantities of soil and field-tested it in three countries.

## Introduction

Soil-transmitted helminths (STH) are a group of intestinal nematodes that include *Ascaris lumbricoides*, *Trichuris trichiura*, and the hookworm species, *Necator americanus* and *Ancylostoma duodenale*. STH infections are one of the most common infections among humans, affecting over 1.5 billion individuals globally, with children and pregnant women at highest risk for associated morbidity [1]. STH are often endemic in low-income countries of Asia and Africa, where centralized or improved sanitation infrastructure remains limited in access [2]. Infection occurs through ingestion of fully developed embryonated eggs of *A*. *lumbricoides* and *T*. *trichiura* or larval penetration of the skin by hookworm larvae present in contaminated soil; *A*. *duodenale* larvae can also rarely infect through ingestion [3].

The primary strategy to date for controlling morbidity associated with STH infections in endemic settings is preventive chemotherapy for high-risk groups with albendazole or mebendazole either annually or biannually [4]. However, in settings with low coverage of networked sanitation and water supply infrastructure, persistent environmental reservoirs of STH eggs likely limit the effectiveness of these intervention programs through increased chance of reinfection [5–8]. A meta-analysis of studies from settings with medium-to-high endemic STH prevalence identified an average 12-month reinfection rate for *A*. *lumbricoides*, *T*. *trichiura*, and hookworm of 94%, 82%, and 57%, respectively [9]. Recent evidence suggests that community-wide mass drug administration (cMDA) can interrupt STH transmission when compared to targeted deworming [10,11]. The success of these intervention programs relies on many factors like coverage, safely managed sanitation access, and other forms of infrastructure and economic development [5,9,12].

**Fig 1:**
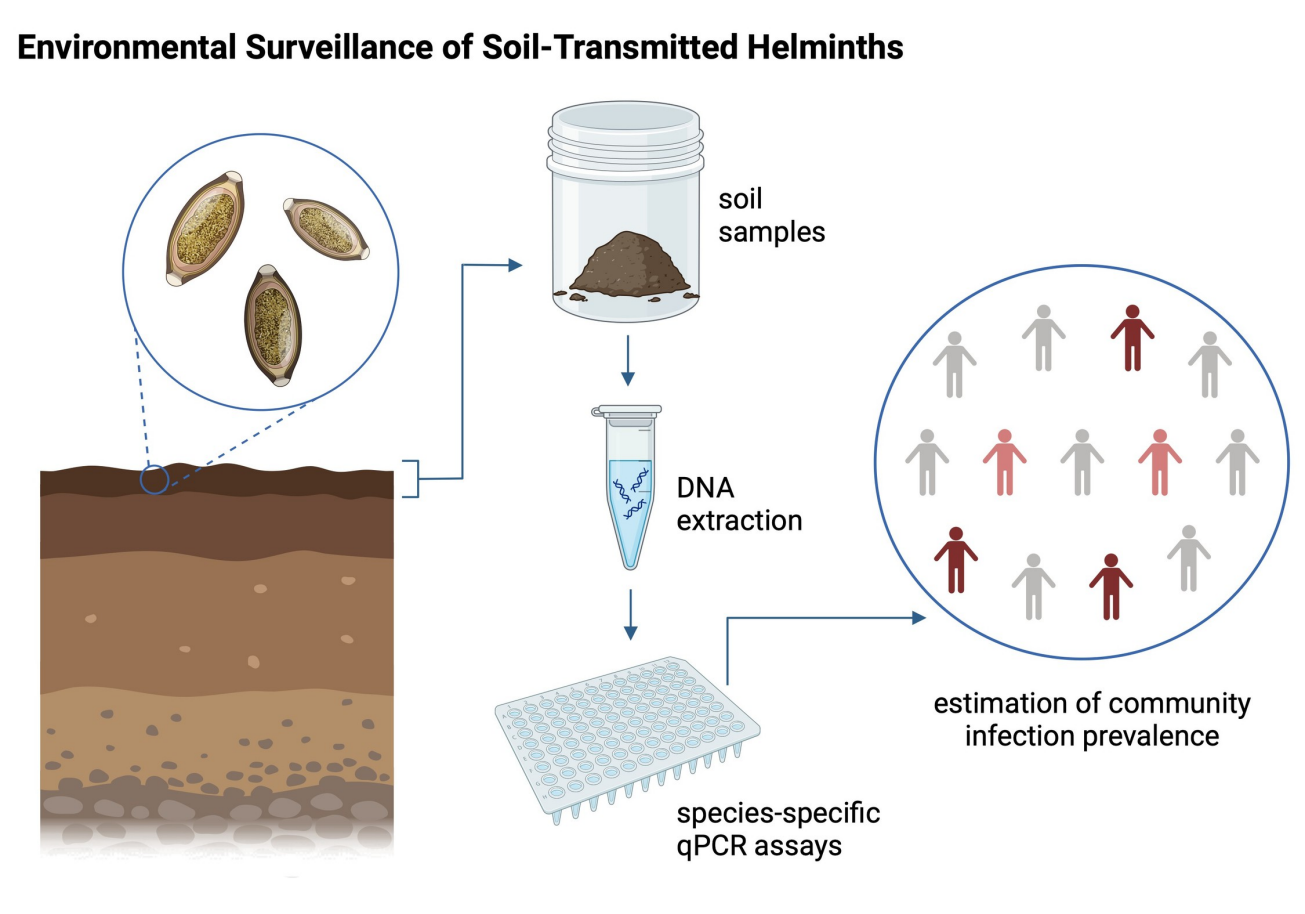
Conceptual figure describing a broad approach for environmental surveillance to monitor soil-transmitted helminth infections. Created with *BioRender*.*com*.

Most MDA control programs continue to rely on surveillance of human stool to assess STH prevalence within communities, however sampling stool from individuals is resource-intensive and logistically challenging to conduct. Recent successes with environmental surveillance for infectious diseases (e.g. COVID, influenza) [13,14] motivates the development of environmental sampling strategies for STH surveillance. Considering that STH spend part of their life cycle in soil, measuring STH eggs in environmental soil in endemic communities could provide valuable data for targeting MDA programs or mathematical modeling of MDA program effectiveness [12,15]. Developing specific and sensitive assays for detecting STH DNA in the soil is a critical first step toward exploring if soil sampling could be a more cost-effective approach for monitoring STH human infection prevalence, especially in resource-limited settings where extensive stool-based surveillance can be challenging (Fig 1). Improved STH surveillance in the context of MDA programs can help target programs to geographic areas where they are needed most, inform when MDA is no longer needed, and trigger additional MDA given early warning signs of recrudescence in the environment.

In recent years, quantitative polymerase chain reaction (qPCR) methods have achieved improved sensitivity for detecting STH infections in stool samples, [16] and have also been used to speciate eggs found in stool and soil samples. Additionally, qPCR assays can be designed to be species-specific and can, therefore, exclude STH that infect animal hosts but are morphologically similar by microscopy [17,18]. Limited studies have compared microscopy and PCR for the detection and speciation of STH in soil. Studies in Kenya and Ethiopia that compared microscopy based on the straining-flotation method and qPCR found microscopy to be more sensitive for *Ascaris* and qPCR to be more sensitive for *Trichuris*, assuming the specificity to be 100% [16,19].

Here we optimize an STH DNA extraction protocol for large-quantity soil samples (20 g) and compare the performance of STH assays using qPCR. Additionally, a subset of samples were tested by droplet digital PCR (ddPCR), which could have better quantification accuracy compared to qPCR. Some studies have also reported lower inhibition with ddPCR, which is helpful for application to soil samples [20,21]. Comparing these two techniques will be useful for selecting the best platform for future environmental STH surveillance protocols. We also field-test our method on soil samples collected from household entrances and household drinking water sources in Benin, Kenya, and India, as well as compare our method to a previously published soil microscopy protocol to detect STH eggs [22]. Further, we assess if STH prevalence in household soil reflects household STH infection prevalence by collecting stool samples from matched households in each study site.

## Methods

### Ethics statement

Written informed consent was obtained from the head of the household or other adult with the ability to make decisions representing the household. Parental or guardian written consent was obtained for child participation. Institutional Review Board (IRB) approval was obtained from Christian Medical College, Vellore (India) (IRB Min no. 10392 dated 08.01.2018), the Ministry of Health in Benin (No. 15/MS/Dc/SGM/DRFMT/CNERS/SA), Kenya Medical Research Institution (KEMRI) Scientific and Ethics Review Unit (Protocol No. 3823) (Kenya), and Tufts Health Sciences Institutional Review Board (#13205).

### Study sites

This study was carried out at three sites: a rural commune in Comé, Benin, the rural health block of Timiri in Ranipet district of Tamil Nadu, India, and urban sub-counties of Kibra and Dagoretti South in Nairobi, Kenya. In each site, we aimed to enroll approximately 100 households for soil and human stool collection. The Benin and India sites were control clusters enrolled in an ongoing cluster randomized trial testing the feasibility of interrupting transmission of STH through expanded cMDA coverage (DeWorm3) [23,24]. The DeWorm3 study sites in Benin and India were previously censused, GIS mapped and divided into clusters, which were randomly assigned to control and treatment arms. Control arms received standard-of-care, school-based deworming (annual in Benin and bi-annual in India) and the intervention arms received bi-annual community-wide deworming with door-to-door drug distribution. This study leveraged the longitudinal monitoring cohort (LMC) in DeWorm3 which consisted of approximately 150 individuals per cluster from whom stool samples were collected annually. Among households with at least two LMC participants in control clusters, approximately 100 households were selected each in India and Benin during this study’s second year of sample collection. In Kenya, sample collection also supported a separate study investigating transmission of *Escherichia coli* across humans, poultry, and the environment [25]. Eligible households had at least one child under 5 years old. The field team systematically approached households for inclusion in the study starting at a compound on a street known to have poultry in the vicinity; only one household was enrolled per compound. After enrolling a household, the field team walked to the next available compound and screened households as needed to ensure an equal number of households with and without poultry were enrolled on each street.

### Survey and sample collection

Household level surveys carried out at the time of sample collection captured data on socio-economic status (SES), access to safe water and sanitation, education, presence of animals (dogs, poultry, or ruminants), and deworming status. All data collection was carried out electronically with Android phones or Samsung tablets using SurveyCTO software (Dobility Inc.). In India and Benin, human stool samples were collected from individuals randomly selected into the LMC (the number of individuals ranged from one to four per household). In Kenya, up to three human stool samples were collected from the following age groups: 0–4, 5–14 and 15+ years. If it was not possible to collect a stool sample from each age group, the team collected either one additional stool sample from children 0–4 years of age or one additional stool sample from children 5–14 years of age. Households were visited up to three times to collect human stool samples. Soil samples were collected immediately outside the household entrance (within 2 m). Soil samples were also collected within 2 m of the household’s reported primary drinking water source; only one sample was collected if multiple enrolled study households used the same water source. Using a stencil to mark off an area of 25 cm x 50 cm, approximately 100 g of soil was collected with a sterile scoop by scraping the top layer of the dirt inside the sampling area moving once vertically and then once horizontally. Both soil and human stool samples reached the laboratory within 3–4 hours of collection and were transported on ice packs.

### Sample processing and physical soil characteristics

Once soil samples reached the laboratory, they were sieved through a screen to remove larger particles and then divided into three aliquots: 1) 20 g for DNA extraction (stored at -80°C); 2) 30 g for soil type, pH, and moisture content measurement (stored at 4°C); and 3) 15 g for soil microscopy (stored at 4°C). Soil pH was measured with a portable pH meter (Fisherbrand accumet AP110) after mixing 5 g of soil with 5 mL of distilled water and incubating at room temperature overnight. Soil moisture content was measured by weighing a soil sample, with an initial mass of 25 g, prior to and after placement in a hot air oven at 110°C for 16 hours. Soil type was categorized based on the ability to form a ball, ribbon and length of the ribbon formed using the same oven-dried soil sample mixed with a small amount of water [26].

Human stool samples were mixed well and then aliquoted by weighing out 500 mg of feces and placing it into a 2 mL cryovial containing 1 mL of 95% or 100% ethanol, followed by vortexing to homogenize. Stool samples were stored at -80°C until DNA extraction.

### Soil microscopy

The soil samples were subjected to a series of filtration and flotation steps to concentrate any eggs present using a previously published protocol [22]. A 50 mL centrifuge tube containing 15 g of a sieved soil sample was filled to the 40 mL mark with 1% 7X solution (MP Biomedicals). The sample was then mixed well and incubated at room temperature overnight. Following incubation, each sample was vortexed and sieved (50 mesh, 300 μm, H&C sieving systems). The sieve was rinsed with 1% 7X solution and approximately 150 mL of the collected flow-through was allowed to settle at room temperature for 30 min. The supernatant was removed and the sediment was evenly divided into two 50 mL centrifuge tubes. The volume in each tube was then increased to 40 mL with 1% 7X solution and samples were centrifuged at 1000 x g for 10 min. Following centrifugation the supernatant was discarded. Five mL of zinc sulfate solution (1.25 specific gravity, flotation solution) was then added to each tube and samples were vortexed and centrifuged at 1000 x g for 5 min. The supernatant collected from both tubes was then combined and sieved (500-mesh sieve, 25 μm, H&C sieving systems). The sediment on the sieve was washed off into a tube with approximately 10 mL of distilled water. The tube was centrifuged, and the supernatant was discarded. Zinc sulfate was again added to the sediment, vortexed, and sieved, and the sediment on the sieve was washed with distilled water. Following this second flotation, the recovered solution was centrifuged at 1000 x g for 5 min and the supernatant was aspirated, leaving a 1 mL volume at the bottom of the tube. This entire 1 mL concentrate was then transferred to a Sedgewick Rafter slide (SPI supplies) and screened at 10X magnification. The morphology of the eggs identified was recorded and the number of eggs counted. If any STH eggs were putatively identified by microscopy, then the contents of the slide were washed back into a centrifuge tube using 1 mL of distilled water. Four mL of 0.1N sulphuric acid was then added to the tube and the sample was incubated at room temperature for 28 days. After 28 days the solution was centrifuged at 1000 x g for 2 min and the supernatant was aspirated, leaving 1 mL of the solution at the bottom of the tube. This residual volume was then screened for larvae to determine the viability of the eggs.

### Molecular analyses

#### Soil DNA extraction

Our goal was to develop a method that would enable processing a large quantity of soil to increase the chance of detecting DNA from STH eggs that can be present at concentrations <1 egg per gram of soil. The Qiagen DNeasy PowerMax Soil Kit was chosen based on the recommended input quantity of up to 10 g of soil. To further increase sensitivity, we modified the protocol to accommodate an initial homogenization and lysis step with 20 g of soil. Briefly, following the addition of soil samples to tubes containing PowerBead solution, the duration of homogenization was increased to 30 min on a vortexing platform; half of this solution was then processed (and the remaining half was discarded). An additional modification included re-loading and repeat centrifugation of extraction products following their final elution from Maxi Spin Columns. This post-elution re-exposure to the column was intended to maximize product recovery. We also modified the manufacturer’s protocol to include the addition of 100 pg of a previously described internal amplification control (IAC) plasmid [27]. This plasmid was added to each sample following the addition of Solution C4 as a process control and to check for PCR inhibition.

Following isolation, samples underwent ethanol precipitation to further purify and concentrate the recovered DNA. To do so, 5 μL of Pellet Paint NF Co-precipitant (MilliporeSigma, Burlington, MA), 500 μL of 3M sodium acetate, and 10 mL of cold 100% ethanol were added to each elution product. Samples were vortexed briefly, incubated at room temperature for 2 min, and then centrifuged at maximum speed with the following conditions; 7,197 x g for 5 min in India, 8,500 x g for 5 min in Benin and 4,472 x g for 10 min in Kenya. Supernatant was then decanted, and 10 mL of cold 70% ethanol was added to each sample. Samples were again vortexed and centrifuged at maximum speed for 5 min. A second wash, this time using cold 100% ethanol, was then performed in an identical fashion. Following the aspiration of ethanol, pellets were allowed to air dry overnight, followed by resuspension in 200 μL of nuclease-free water.

All DNA extractions occurred in the countries in which the samples were collected. A reagent-only “extraction blank” sample was extracted after every 24 soil samples processed. The full extraction protocol is provided in Appendix E in S1 Text.

#### Establishing limits of detection

Limits of detection (LOD) were established at Smith College using locally obtained, non-sample soil of two different types (sandy and organic/loamy soil). To determine LODs for the qPCR-based detection of STH eggs in soil samples, 20 g aliquots of soil were spiked with either 200, 100, 50, 20 10, 5, or 2 *A*. *lumbricoides*, *N*. *americanus*, or *T*. *trichiura* eggs. Eggs were titrated from liquid suspensions with known concentrations. Following the addition of eggs to each sample, the full 20 g mass of each sample was thoroughly homogenized by hand mixing. Samples were processed in triplicate to determine the lowest spiking concentration that could be detected. DNA was extracted using the protocol described above for limit of detection testing.

#### Stool DNA extraction

DNA was extracted from stool samples using the MP Biomedicals FastDNA SPIN Kit for soil (MP Biomedicals) and a FastPrep benchtop homogenizer (MP Biomedicals) following a modified version [28] of a previously published protocol [29]. The same internal amplification control described in the soil sample extractions above was spiked in after lysis (100 pg IAC) [27].

#### Multi-parallel qPCR

Previously published multi-parallel qPCR assays targeting non-coding repetitive sequences were utilized to detect *N*.*americanus*, *T*.*trichiura*, [30] and *A*.*lumbricoides* [31] in both soil and stool samples **(**Table H in S1 Text**)**. We developed a new assay to detect *A*. *duodenale* (see Tables I and J in S1 Text for validation results). Stool samples from India and all soil samples were additionally tested using a previously published assay for the presence of *Ancylostoma ceylanicum*, [32] a zoonotic species of hookworm known to contribute to human infection in many parts of Asia [33]. Further details on assay choice and development can be found in Appendix A in S1 Text.

All samples were tested in duplicate and a titration of plasmid (10 pg, 100 fg, and 1 fg) containing a single copy of the target sequence for each assay was utilized as a positive PCR control. ‘No template control’ samples were also tested on each qPCR reaction plate. For all the STH assays, cycling conditions included an initial 2 min incubation step at 50°C, followed by a 10 min incubation at 95°C, then 40 cycles of 15 sec at 95°C for denaturation and 1 min at 59°C for annealing and extension. The detailed protocol can be found in Appendix F in S1 Text. All qPCR reactions were carried out using the Quantstudio 7 Flex PCR system (Applied Biosystems) and the data generated was analyzed using Quantstudio Real-Time PCR software Version 1.3. A sample with a Cq value <40 in both replicates was reported as positive for the target tested. A sample returning a positive result in only one of two test replicates was re-tested, again in duplicate, and was reported positive only if the second testing had at least one positive replicate with a Cq value <40. If the IAC failed in qPCR, the sample was re-extracted; if the IAC failed again after re-extraction, the sample was excluded from analyses. In all cases of re-testing, the Cq value of the re-test was used in the analysis. We tested for inhibition using the *N*. *americanus* assay, described in detail in Appendix B in S1 Text.

#### Droplet digital PCR

A subset of 50 randomly selected soil DNA aliquots from Benin, India and Kenya were tested for *N*. *americanus*, *A*. *lumbricoides*, and *T*. *trichiura* by ddPCR at Christian Medical College, Vellore. All primers and probes were identical to those described above for qPCR and reactions were performed using the QX200 Droplet Digital PCR system (Bio-Rad). The ddPCR reaction mix for each target assay consisted of 11 μL of 2x ddPCR Supermix for Probes (Bio-Rad); primers (250 nM concentrations of each primers for *N*. *americanus* and 62.5 nM concentrations for *A*. *lumbricoides* and *T*. *trichiura*); probes (125 nM concentrations for all assays) and 4 μL of sample DNA, resulting in a final reaction volume of 22 μL. Droplets were generated in the QX200 droplet generator (Bio-Rad) with 20 μL of the reaction mix and 70 μL of droplet generating oil in an 8 channel DG8 cartridge. Droplets in oil suspensions were transferred to a 96-well semi-skirted ddPCR plate (Bio-Rad) and placed into a C1000 Touch Thermal Cycler (Bio-Rad). Cycling conditions included an initial denaturation step at 95°C for 10 min, followed by 40 cycles of 94°C for 30 sec and 59°C for 1 min. Cycling was followed by a final hold at 98°C for 10 min. Droplets were read automatically by the QX200 droplet reader (Bio-Rad) and the data was analyzed with the QuantaSoft Version 1.7.4 (Bio-Rad). Any sample with 3 or more droplets at least in one well was considered positive [34–36].

### Statistical analyses

To evaluate the agreement between ddPCR and qPCR, and between microscopy and qPCR for the detection of STH in soil, we calculated the percent agreement as the number of samples for which the two methods agreed (either positive/positive or negative/negative), divided by the total number of samples tested. We then estimated the Kappa statistic, with asymptotic standard errors and p*-*values using an alpha of 0.05 for statistical significance, to determine whether agreement was poor (*κ* < 0), slight (0.01–0.20), fair (0.21–0.40), moderate (0.41–0.60), substantial (0.61–0.80), or perfect (0.81–1.00) [37].

We estimated bivariate associations between characteristics of soil samples and detection of STH in soil samples using logistic regression models. Outcome variables were presence/absence for each STH target in each soil sample, detected via qPCR. Soil characteristics of interest included soil sampling location, soil type, moisture content, shade/sun, presence of feces, and pH; these variables are further described in **Table D in S1 Text**. We used generalized estimating equations (GEE) with an exchangeable working correlation to estimate robust standard errors and adjust for repeated soil samples at the household level.

We also estimated associations between household-level STH prevalence in soil and stool from matched households for each STH target, detected by qPCR. Soil characteristic variables screened for associations with STH in soil were included as covariates in these models if associations were statistically significant, using a cutoff of *p* < 0.20. Outcome variables were presence/absence for each STH target at the household level (i.e., whether any stool sample from that household was positive). For this analysis, we included only households where both soil and stool samples were successfully collected. We also only included STH targets with a household-level stool prevalence of > 5% at a given study site to avoid positivity assumption violations due to low outcome prevalence. We report both unadjusted and adjusted associations, where adjusted models included covariates after variable selection for each STH outcome, described above. Study site (country) was also included as a covariate in all adjusted models. We used GEE with an exchangeable working correlation and a Poisson distribution due to the zero-inflated nature of the outcome data to avoid model convergence issues. We report measures of association as odds ratios (OR) with 95% confidence intervals (CI). We used R (V 1.0.143) for all tables, figures, and statistical analyses using packages *tidyr*, *arsenal*, *ggplot2*, *vcd*, and *geepack* [38–43].

## Results

### Household and soil sample characteristics

In total, we analyzed 478 soil samples and 669 stool samples for STH across 322 households in Benin, India, and Kenya. Household drinking water sources varied by country, though most households had access to an improved drinking water source **(Table 1)**. Public taps/standpipes were one of the most common water sources in Benin (61%), India (39%), and Kenya (69%). Other common water sources included unprotected dug wells in rural Benin (24%) and tube wells or boreholes in urban Kenya (21%); nearly half of households in India had piped water into the household (49%), compared to almost no households in Benin or Kenya **(Table 1)**.

**Table 1:** Household characteristics in each study site and overall.

	Benin (rural) n (%)	India (rural) n (%)	Kenya (urban) n (%)	Total n (%)
Total Households	104 (100)	99 (100)	119 (100)	322 (100)
Total Soil Samples				
*Household entrance*	104 (100.0)	99 (100)	117 (98.3)	320 (99.4)
*Household water source*	56 (53.8)	54 (54.5)	48 (40.2)	158 (49.0)
Drinking water collection location				
*Directly from a filter*	0 (0.0)	0 (0.0)	5 (4.2)	5 (1.6)
*Directly from storage container*	52 (50.0)	91 (91.9)	114 (95.8)	257 (79.8)
*Directly from water source*	52 (50.0)	8 (8.1)	0 (0.0)	60 (18.6)
Improved drinking water source				
*Public tap/standpipe*	60 (61.2)	35 (38.9)	81 (69.2)	176 (57.7)
*Tube well or borehole*	7 (7.1)	1 (1.1)	24 (20.5)	32 (10.5)
*Piped into dwelling*	1 (1.0)	44 (48.9)	0 (0.0)	45 (14.8)
*Piped to yard/plot*	5 (5.1)	6 (6.7)	7 (6.0)	18 (5.9)
*Protected dug well*	0 (0.0)	4 (4.4)	0 (0.0)	4 (1.3)
Unimproved drinking water source				
*Unprotected dug well*	23 (23.5)	0 (0.0)	0 (0.0)	23 (7.5)
*Cart with small tank or tanker truck*	0 (0.0)	0 (0.0)	4 (3.4)	4 (1.3)
*Unprotected spring*	2 (2.0)	0 (0.0)	0 (0.0)	2 (0.7)
*Other*	0 (0.0)	0 (0.0)	1 (0.9)	1 (0.3)
*N-Missing*	6	9	2	17
Household drinking water treatment				
*No*	96 (95.0)	79 (86.8)	97 (82.9)	272 (88.0)
*Yes*	5 (5.0)	12 (13.2)	20 (17.1)	37 (12.0)
*N-Missing*	3	8	2	13
Water storage container				
*Drum (metal/plastic) with lid*	13 (12.9)	5 (5.5)	0 (0.0)	18 (5.9)
*Drum (metal/plastic) without lid*	2 (2.0)	0 (0.0)	0 (0.0)	2 (0.7)
*Jerrycan (metal/plastic)*	6 (5.9)	0 (0.0)	65 (56.5)	71 (23.1)
*Plastic tub or bucket with lid*	9 (8.9)	0 (0.0)	34 (29.6)	43 (14.0)
*Plastic tub or bucket without lid*	0 (0.0)	0 (0.0)	1 (0.9)	1 (0.3)
*Water or cooking pot (plastic/metal/clay)*	71 (70.3)	86 (94.5)	8 (7.0)	165 (53.7)
*Water storage vessel with lid*	0 (0.0)	0 (0.0)	4 (3.5)	4 (1.3)
*Other*	0 (0.0)	0 (0.0)	3 (2.6)	3 (1.0)
*N-Missing*	3	8	4	15
Water use method				
*Container/glass dipped into water container*	99 (98.0)	91 (100.0)	29 (24.8)	219 (70.9)
*Ladle used to obtain water*	0 (0.0)	0 (0.0)	7 (6.0)	7 (2.3)
*Water poured from container*	2 (2.0)	0 (0.0)	75 (64.1)	77 (24.9)
*Water poured from tap/handpump*	0 (0.0)	0 (0.0)	6 (5.1)	6 (1.9)
*N-Missing*	3	8	2	13
Owns poultry	80 (76.9)	17 (17.2)	58 (48.7)	155 (48.1)
Owns dogs	32 (30.8)	4 (4.0)	6 (5.0)	42 (13.0)
Owns ruminants	58 (56.3)	42 (42.4)	2 (1.7)	102 (31.8)
*N-Missing*	1	0	0	1

Of 478 soil samples, 67% were collected from the household entrance while 33% were collected at the household water source **(Table 2)**. Soil samples were most frequently classified as sand (32%) or loamy sand (18%) in Benin, sandy loam (47%) or loam (33%) in India, and loam (17%), sandy loam (14%), clay loam (14%), or sand (14%) in Kenya. Feces was visible near 27% of all soil sampling locations across the different study sites. Soil moisture content was highest on average in Kenya (mean: 22%, standard deviation (SD): 12%), while soil pH was highest in Benin (mean: 8.06, SD: 0.34) **(Table 2)**. Moisture content was also slightly higher in soil collected from household water sources (mean: 13.30, SD: 10.87) compared to soil from the household entrance (mean: 10.22, SD: 11.66) **(Table E in S1 Text).**

**Table 2:** Characteristics of soil samples collected in each study site and overall.

	Benin n (%)	India n (%)	Kenya n (%)	Total n (%)
Total Samples	160 (100)	153 (100)	165 (100)	478 (100)
Sample Type				
*Household entrance soil*	104 (65.0)	99 (64.7)	117 (70.9)	320 (66.9)
*Water source soil*	56 (35.0)	54 (35.3)	48 (29.1)	158 (33.1)
Soil Type A (most stable)	63 (39.4)	69 (45.1)	101 (61.2)	233 (48.7)
*Clay*	3 (1.9)	1 (0.7)	6 (3.7)	10 (2.1)
*Clay loam*	19 (11.9)	9 (5.9)	22 (13.6)	50 (10.5)
*Loam*	14 (8.8)	51 (33.3)	27 (16.7)	92 (19.4)
*Sandy clay*	2 (1.2)	0 (0.0)	0 (0.0)	2 (0.4)
*Sandy clay loam*	11 (6.9)	3 (2.0)	13 (8.0)	27 (5.7)
*Silty clay*	0 (0.0)	1 (0.7)	11 (6.8)	12 (2.5)
*Silty clay loam*	14 (8.8)	4 (2.6)	22 (13.6)	40 (8.4)
Soil Type B	17 (10.6)	84 (54.9)	36 (21.8)	137 (28.7)
*Sandy loam*	10 (6.2)	72 (47.1)	23 (14.2)	105 (22.0)
*Silt loam*	7 (4.4)	12 (7.8)	13 (8.0)	32 (6.7)
Soil Type C (least stable)	80 (50.0)	0 (0.0)	25 (15.2)	105 (22.0)
*Loamy sand*	29 (18.1)	0 (0.0)	3 (1.9)	32 (6.7)
*Sand*	51 (31.9)	0 (0.0)	22 (13.6)	73 (15.4)
Feces visible at sampling location	44 (27.5)	32 (20.9)	39 (23.6)	115 (24.1)
Soil visibly wet	86 (53.8)	16 (10.5)	63 (38.2)	165 (34.5)
Soil in sun				
*Partly sunny*	23 (14.4)	1 (0.7)	91 (55.2)	115 (24.1)
*Shaded*	28 (17.5)	3 (2.0)	18 (10.9)	49 (10.3)
*Sunny*	109 (68.1)	149 (97.4)	56 (33.9)	314 (65.7)
Soil moisture (%)				
*Mean (SD)*	6.19 (5.55)	5.00 (7.35)	21.92 (11.29)	11.24 (11.48)
*Range*	0.00–19.65	0.00–43.42	0.00–67.21	0.00–67.21
Soil pH				
*Mean (SD)*	8.06 (0.34)	7.81 (0.28)	7.88 (0.46)	7.92 (0.39)
*Range*	6.95–9.35	6.63–8.54	5.66–9.34	5.66–9.35

### STH detection in soil samples by microscopy, qPCR, and ddPCR

Detection limits varied for each helminth species. Through a series of spiking experiments, we determined our new method has a detection limit of five *A*. *lumbricoides* eggs per 20 g of soil (0.25 eggs per gram [EPG] of soil), two hookworm eggs per 20 g of soil (0.1 EPG soil), and ten *T*. *trichiura* eggs per 20 g of soil (0.5 EPG soil).

All extraction blanks (n = 14) and non-template control (NTC) wells (n = 166) were negative for all target STH qPCR assays. All qPCR plates had detection of positive controls for each assay. IAC spiking results are reported in SI (Appendix C in S1 Text). After removing samples without IAC amplification for analysis, our final dataset included 160 soil samples from 104 households in Benin, 152 soil samples from 99 households in India, and 137 soil samples from 102 households in Kenya (449 total soil samples from 305 households).

Field testing of soil in India, Benin, and Kenya demonstrated that STH DNA is frequently detected in soil from households and drinking water sources. By qPCR detection, the overall prevalence of *A*. *lumbricoides* was 31%, *T*. *trichiura* was 3%, and any hookworm species (*N*. *americanus*, *A*. *duodenale*, or *A*. *ceylanicum)* was 13%. *Ascaris* was the predominant STH in soil samples from Benin (26%) and Kenya (59%), while hookworm was the predominant STH in India (18%) **(Table A in S1 Text)**. qPCR detected up to three different hookworm species, with *N*. *americanus* predominant in all three countries **(Table A in S1 Text)**. *A*. *ceylanicum* was assessed by qPCR in all soil samples and in stool samples in India based on previous reports of detection in South Asia; all stool samples were negative and one household water source soil sample was positive. *A*. *duodenale* was assessed by qPCR in all countries but was also only detected in one household water source soil sample. Due to the low prevalence, results for *A*. *duodenale* and *A*. *ceylanicum* are not included in further analyses.

**Fig 2:**
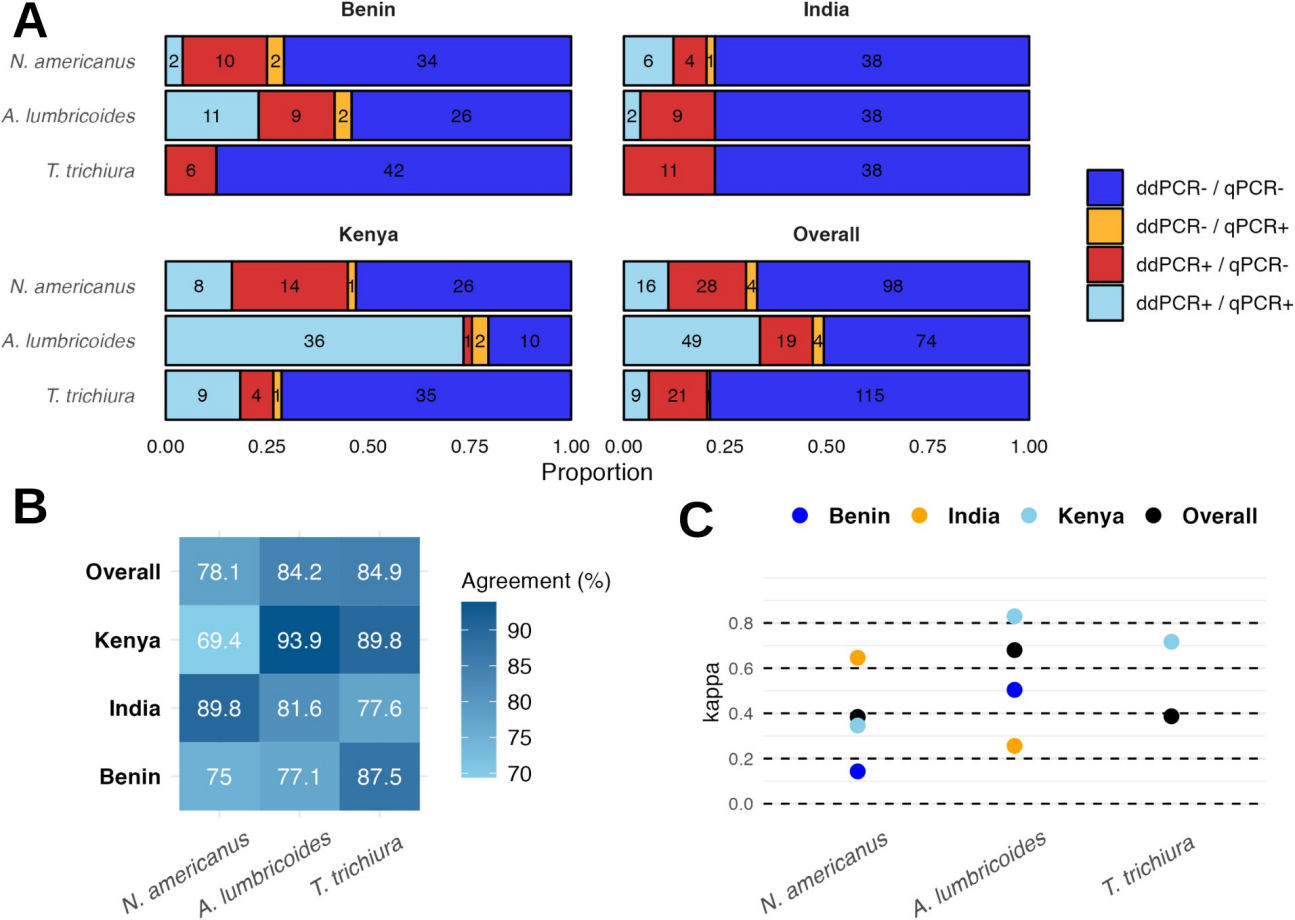
STH detection in soil using qPCR versus ddPCR (A), with percent agreement (B) and Cohen’s Kappa statistic to assess strength of agreement (C). Criteria for positivity by ddPCR was an average of ≥3 positive droplets (2 replicates run).

In comparisons of qPCR versus ddPCR STH detection in soil samples, there was good agreement between the two approaches. We found 78% agreement for *N*. *americanus* detection by qPCR and ddPCR, 84% for *A*. *lumbricoides*, and 85% for *T*. *trichiura* across the study sites **(Fig 2 and Table B in S1 Text)**. Kappa statistics of agreement indicated statistically significant fair to substantial agreement between qPCR and ddPCR STH detection overall for each species **(Fig 2 and Table B in S1 Text)**.

Agreement between microscopy and qPCR for STH detection in soil was lower than agreement between ddPCR and qPCR. Overall agreement across all study sites was 74% for any hookworm, 73% for *Ascaris*, and 81% for *Trichuris* **(Table C in S1 Text)**. Kappa statistics indicated poor agreement, with the highest and only statistically significant Kappa statistic indicating fair agreement for *Ascaris* **(Table C in S1 Text)**. By light microscopy, the prevalence of *Ascaris* was lower than qPCR (20% versus 31%), *Trichuris* was higher (16% versus 3%), and hookworm was substantially lower (6% versus 24%) in soil samples **(Table A in S1 Text)**. In Kenya, *Ascaris* was detected in 30% of soil samples using microscopy but was detected in 63% of samples using qPCR, suggesting higher sensitivity by qPCR **(Table C in S1 Text)**. The prevalence of hookworm in soil was substantially underestimated using microscopy versus qPCR in all three study sites (Fig A and Table C in S1 Text), even when including qPCR detects using the non-specific assay for *A*. *duodenale* which also detected *A*. *caninum*. The underestimation of hookworm using microscopy was likely because hookworm degrades during the soil microscopy protocol used in this study, which takes almost 24 hours to complete. By microscopy, *Trichuris* was detected in 11% and 19% of soil samples in India and Benin, but was not detected in any soil samples in India or Benin via qPCR **(Table C in S1 Text)**. *Trichuris* was also at higher prevalence by microscopy in Kenya compared to qPCR.

Bivariate associations between soil characteristics and detection of STH in soil by qPCR varied based on the target STH species **(Table 3)**. Samples classified as soil Type C–the least stable soil type including sand and loamy sand–had lower odds of *N*. *americanus* detection (OR: 0.48, 95% CI: 0.21, 1.12) **(Table 3)**. Exposure to full sun was associated with lower odds of *A*. *lumbricoides* detection in soil (OR: 0.17, 95% CI: 0.10, 0.29), while the odds of *A*. *lumbricoides* detection was two times higher if the soil was wet at the time of sample collection (OR: 2.29, 95% CI:1.44, 3.63) **(Table 3)**. *T*. *trichiura* detection in soil was more than four times as likely if the soil was wet (OR: 4.37, 95% CI: 1.31, 14.57) **(Table 3)**.

**Table 3:** Bivariate associations between soil characteristics and soil-transmitted helminth (STH) detection in soil samples (n = 449) by qPCR.

	Unadjusted Odds Ratio (95% CI)			
	*A*. *lumbricoides*	*N*. *americanus*	*T*. *trichiura*	Any STH^a^
Water source soil (ref = Household soil)	1.42 (0.96, 2.09)*	0.80 (0.43, 1.49)	2.27 (0.75, 6.81)*	1.13 (0.76, 1.69)
Soil Type B (ref = Type A)	0.68 (0.41, 1.13)*	1.10 (0.60, 2.03)	0.63 (0.16, 2.41)	0.79 (0.50, 1.26)
Soil Type C (ref = Type A)	1.23 (0.72, 2.11)	0.48 (0.21, 1.12)*	0.51 (0.11, 2.45)	1.04 (0.43, 1.18)
Soil Moisture Content (10% increase)	1.007 (1.005, 1.009)**	0.998 (0.995, 1.001)*	1.009 (1.005, 1.014)**	1.004 (1.002, 1.006)**
Soil pH (1-unit increase)	1.32 (0.70, 2.48)	0.62 (0.30, 1.31)	0.33 (0.09, 1.23)*	1.13 (0.64, 1.98)
Sample in shade (ref = Partial shade)	0.53 (0.22, 1.27)*	0.73 (0.19, 2.77)	3.04 (0.75, 12.33)*	0.62 (0.26, 1.44)
Sample in sun (ref = Partial shade)	0.17 (0.10, 0.29)**	1.56 (0.73, 3.36)	0.32 (0.08, 1.29)*	0.31 (0.19, 0.51)**
Sample visibly wet (ref = Dry)	2.29 (1.44, 3.63)**	0.70 (0.38, 1.31)	4.37 (1.31, 14.57)**	1.73 (1.13, 2.63)**
Feces visible at sampling location (ref = No)	1.43 (0.86, 2.38)*	1.12 (0.56, 2.25)	1.98 (0.62, 6.25)	1.47 (0.93, 2.33)*

^a^Any STH excluding *A. duodenale* and *A. ceylanicum* due to low prevalence in soil.

CI: confidence interval; STH: soil-transmitted helminth.

*Indicates *p* < 0.2 (cutoff for inclusion in regressions estimating association between STH in soil and in matched stool samples). **Indicates statistical significance given *p* < 0.05. Soil Type A: Clay, Clay loam, Loam, Sandy clay, Sandy clay loam, Silty clay, Silty clay loam; Soil Type B: Sandy loam, Silt loam; Soil Type C: Loamy sand, Sand.

### qPCR detection of STH in household-matched soil and stool samples

We assessed STH prevalence in 669 human stool samples in Benin (N = 248), India (N = 142), and Kenya (N = 279) matched to soil samples collected from the same households. *A*. *lumbricoides* was detected in individual stool samples from Benin (5%) and Kenya (25%), though it was not detected in any samples in India **(Table A in S1 Text)**. Kenya had the highest infection prevalence for *T*. *trichiura* (5.4%), while India had the highest prevalence of *N*. *americanus* (19%) **(Table A in S1 Text)**. Overall, 31.9% of households (n = 307) had at least one stool sample that was positive for any given STH species **(Table 4)**. STH were detected more frequently in soil from household water sources (40.0%) and household entrances (38.4%). *A*. *lumbricoides* was the most frequently detected STH species in soil at household entrances (28.6%), in soil at water sources (34.8%), and in humans (17.6%) **(Table 4)**. STH prevalence was similar in soil from the household entrance and in soil from household water sources across all STH species **(Table 4)**.

**Table 4:** Overall household-level prevalence of soil-transmitted helminths (STH) detected by qPCR in soil and stool across all study sites in Benin, India, and Kenya.

	Household Water Source Soil (N = 155) n (%)	Household Entrance Soil (N = 294) n (%)	Household Stool^a^ (N = 307) n (%)
*A*. *lumbricoides*	54 (34.8)	84 (28.6)	54 (17.6)
*N*. *americanus*	17 (11.0)	39 (13.3)	43 (14.0)
*A*. *duodenale*	1 (<1)	0 (0)	0 (0)
*A*. *ceylanicum*^*b*^	1 (<1)	0 (0)	0 (0)
*T*. *trichiura*	7 (4.5)	7 (2.4)	15 (4.9)
Any STH	62 (40.0)	113 (38.4)	98 (31.9)

^a^ For stool samples, household-level prevalence was determined based on whether any stool samples (of up to 4) collected from a household were positive for a given STH target.

^b^*Assay was only tested on samples from India*.

**Fig 3:**
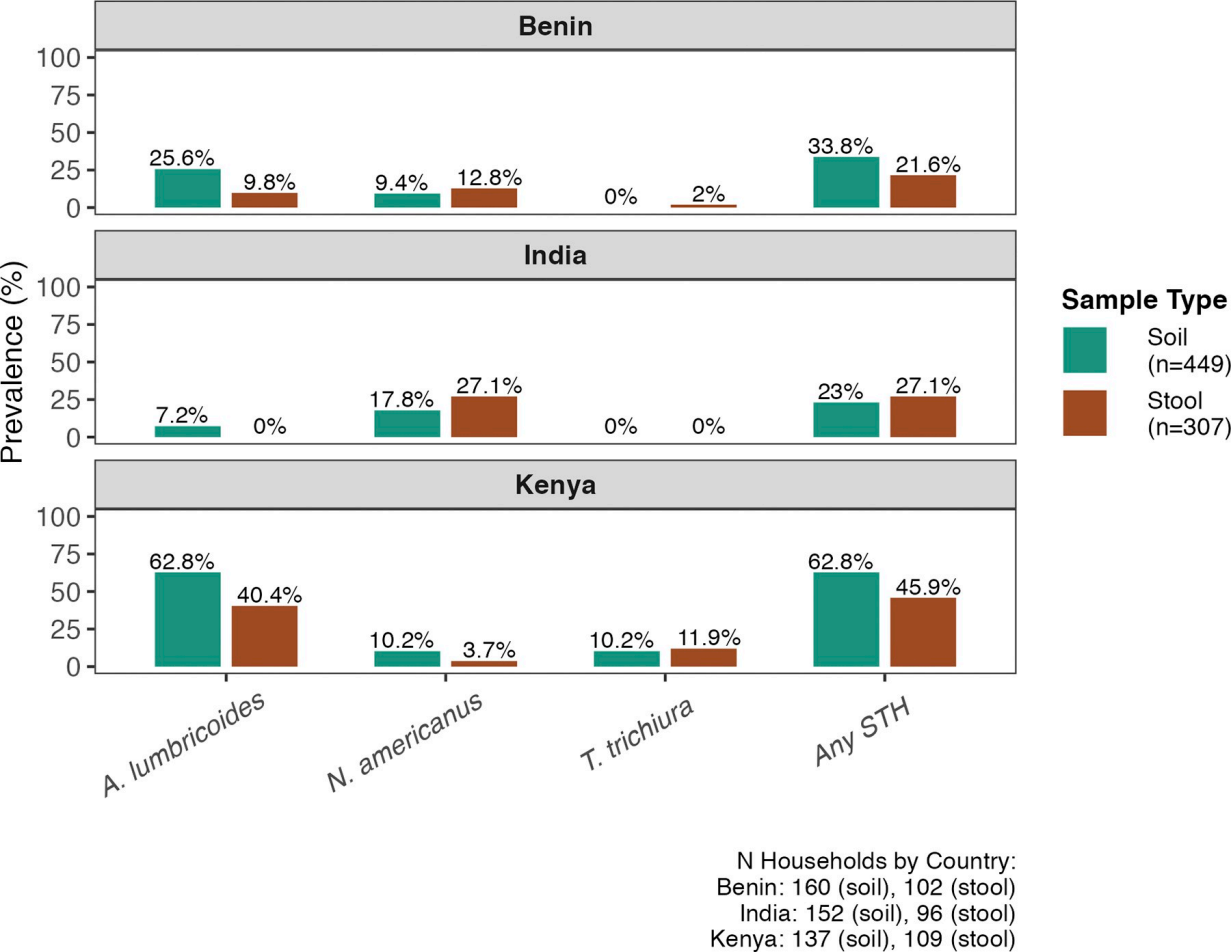
Household-level prevalence of *A. lumbricoides, N. americanus, T. trichiura*, and any soil-transmitted helminths (STH) by qPCR detection, stratified by country and sample type. For stool samples, household-level prevalence is determined based on whether any stool samples (of up to 4) collected from a household were positive for a given STH target. *A. duodenale* and *A. ceylanicum* are not included due to low prevalence in soil and stool across all three countries.

In matched stool and soil samples, soil STH profiles via qPCR detection typically reflected stool STH infection profiles across the study sites. *A*. *lumbricoides* was the most frequently detected STH target in soil samples (62.8%) and in stool from matched households (40.4%) in Kenya **(Fig 3)**. In India, *N*. *americanus* was most frequently detected in stool (27.1% of households), with a similar detection frequency in soil (17.8%) **(Fig 3)**. We observed that even when human infection prevalence is low, STH DNA can still be detected in soil. For example, *N*. *americanus* infection prevalence among the 109 households sampled in Kenya was 3.7%, while the prevalence in soil from matched households was 10.2% **(Fig 3)**.

**Fig 4:**
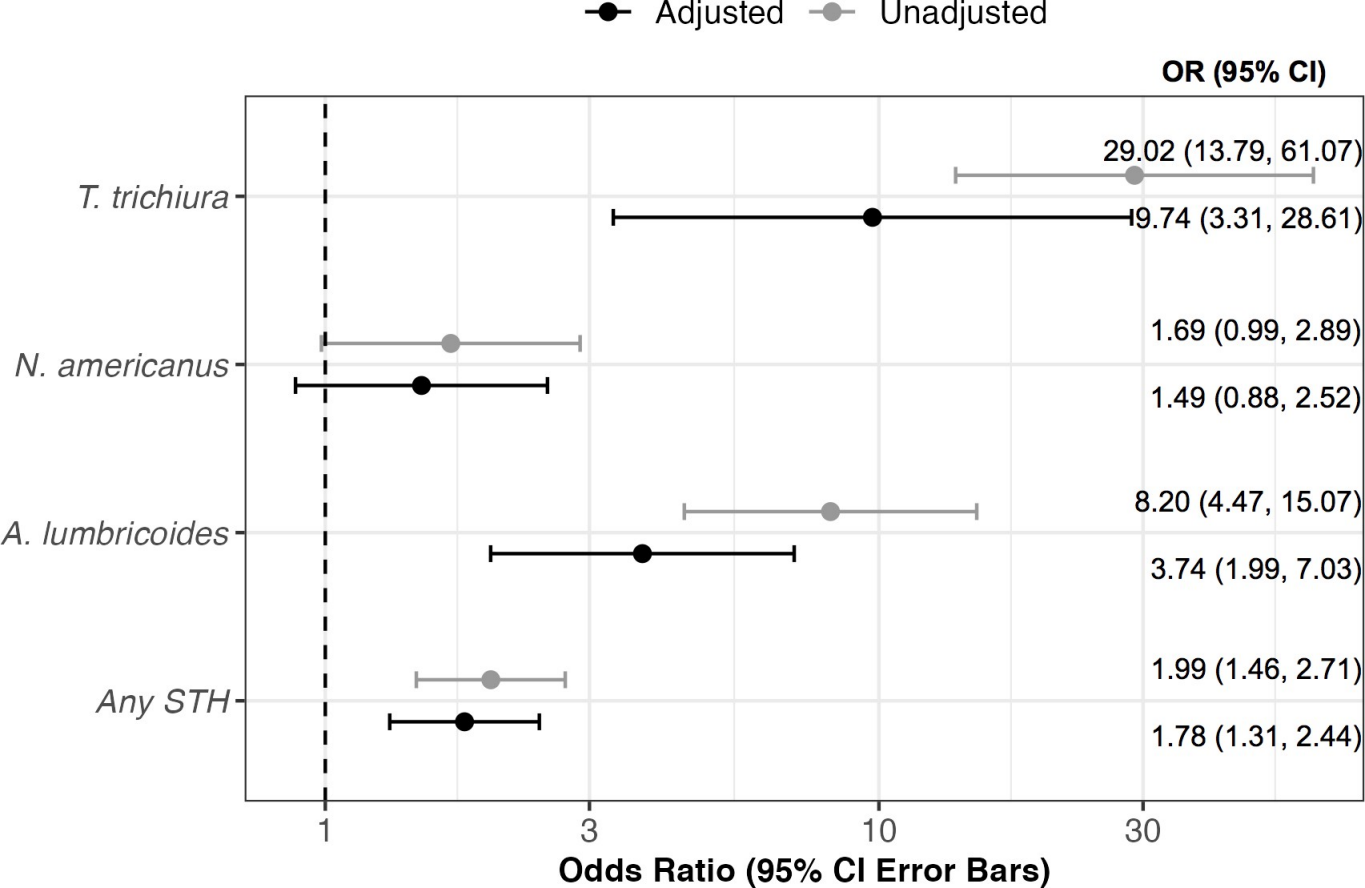
Unadjusted and adjusted associations between soil-transmitted helminth (STH) detection by qPCR in soil and matched human stool samples from 290 households in India, Kenya, and Benin. Points represent odds ratios (OR) with 95% confidence interval (CI) error bars. Any STH indicates the sample was positive for at least one of the following targets: *A. lumbricoides, T. trichiura, or N. americanus*. Adjusted models included covariates associated (*p* < 0.20) with soil STH detection for each target, and all adjusted models including study site (country). Adjusted model for “Any STH” included variables for sun exposure, soil moisture content, whether the sampling area was visibly wet, and whether the sampling area had visible feces nearby; N. americanus adjusted model included soil type and moisture content; A. lumbricoides adjusted model included sample type, soil type, soil moisture, sun exposure, whether the sampling area was visibly wet, and whether there was visible feces; T. trichiura adjusted model included sample type, soil moisture, pH, sun exposure, and whether the sampling area was visibly wet.

STH detection in soil was strongly linked to detection of most STH targets in matched household samples (n = 290 households after removing samples with failed IAC or missing soil characteristic data) with and without adjustment for soil characteristics. The odds of *A*. *lumbricoides* detection in stool was 3.74 times higher given detection in matched household soil (aOR: 3.74, 95% CI: 1.99, 7.03) **(Fig 4)**. The odds of *T*. *trichiura* detection in stool was nearly 10 times higher given detection in matched household soil (aOR: 9.74, 95% CI: 3.31, 28.61), though the estimates were imprecise due to the low prevalence of *T*. *trichiura* **(Fig 4)**. *N*. *americanus* detection in soil was marginally associated with detection in stool from matched households (aOR: 1.49, 95% CI: 0.88, 2.52), though the association was not statistically significant **(Fig 4)**. When considering any STH species (excluding *A*. *duodenale* and *A*. *ceylanicum*), the odds of detection in stool was 1.78 times higher given detection in matched household soil (aOR: 1.78, 95% CI: 1.31, 2.44) **(Fig 4)**. *A*. *duodenale and A*. *ceylanicum were* excluded from the regression analysis given the lack of detection across all three study sites.

## Discussion

Through laboratory and field experiments we optimized a method to process and analyze large quantity (20 g) soil samples for STH DNA. Detection of *T*. *trichiura* was possible at 0.5 EPG of soil, *A*. *lumbricoides* at or above 0.25 EPG soil, and *N*. *americanus* at or above 0.1 EPG soil. The method has several strengths. First, direct extraction of DNA from raw soil does not require lengthy flotation or other egg concentration steps that could result in egg loss [22]. Second, samples can be collected and stored frozen, enabling analysis in batches when convenient. Third, the method is novel in allowing for processing of large quantities of soil (up to 20 g per sample), whereas other soil DNA extraction methods are typically limited to <0.5 g (e.g. Quick-DNA Fecal/Soil Microbe Kits, Zymo Research; DNeasy PowerSoil Pro Kits, QIAGEN). Fourth, using molecular assays allowed us to ensure we were using assays that are specific to relevant STH species that infect humans. The latter is particularly important for environmental surveillance, as soil samples may contain a variety of animal-specific STH that are not relevant for assessing human infection prevalence. Comparing ddPCR and qPCR showed good agreement for STH detection in soil. However, we identified several advantages of qPCR over ddPCR, including reduced variability between replicates **(Table F in S1 Text)**, comparable sensitivity, and lower cost and wider availability of equipment.

Soil characteristics varied within and across our study sites, which can potentially impact STH presence and detection. In microscopy-based studies, recovery rates of hookworm and other STH eggs have been lower in sandy soils compared to clay soils [44–48]. Molecular methods may improve detection of certain STH in a variety of soil types, in this study we did not detect major differences in sandy soil samples compared to other soil types by qPCR. Sun exposure was also negatively associated with detection of *A*. *lumbricoides*, while soil moisture content was positively associated with detection of all STH species, as has been found in a previous study in Kenya using microscopy to detect STH [49]. Given the role of sunlight in desiccation and soil moisture in the growth and activation of STH species, these two variables are potentially important for understanding the role of soil-reservoirs of STH in a community and considerations for soil sampling strategies. While additional data on soil characteristics may be informative for developing sampling strategies, the presence of STH in soil was strongly associated with STH detection in matched stool without adjustment for soil characteristic variables. Collecting and analyzing soil samples alone may be sufficient for predicting STH prevalence at the community level, reducing requirements for field surveys or additional observational data.

We observed higher prevalence of *Ascaris* in soil compared to human stool across the three study sites, while we observed the opposite trend for hookworm in Kenya and Benin (Fig 3). The higher prevalence of *Ascaris* in the soil can be due to its ability to stay intact in the environment for a much longer time, approximately ten years. In contrast, hookworm eggs develop into third stage larvae within a few days and can remain viable in the soil for about 3–4 weeks [22]. These results suggest that species specific relationships between soil prevalence and human infection prevalence are different, and would need to be considered when interpreting soil surveillance data.

While light microscopy protocols for STH detection in soil can be low-cost and avoid the need for expensive equipment, [22] our results suggest that specificity and sensitivity is limited [22]. In our India study site, *Trichuris* detected by light microscopy in 11 soil samples were negative by qPCR, suggesting that these samples may have contained a morphologically similar but different species of *Trichuris* (e.g. *Trichuris ovis* that infects goats). This could also explain the higher prevalence of *Trichuris* in Kenya by microscopy compared to qPCR. Other studies have reported misclassification of human STH in human stool samples through microscopy, with particularly low sensitivity for the detection of hookworm in low prevalence settings [50,51]. The discrepancies between prevalence estimates by microscopy and qPCR highlights the challenges associated with identification of human-specific STH species in soil using microscopy. Protocols for concentrating eggs for light microscopy from soil use a series of sieving, settling, flotation, and centrifugation steps, which can be labor intensive, time consuming, and prone to both human error and egg loss. Highly trained and experienced lab technicians are needed to ensure correct identification of STH eggs in soil samples, as samples can contain many types of non-STH nematode eggs. Additionally, hookworm can be too fragile to withstand the processing time required for soil microscopy (e.g. 24 hours) resulting in false negative samples [22]. For example, studies in rural Kenya and rural Bangladesh that collected and processed > 2,000 soil samples by microscopy did not detect hookworm in any sample [52]. The processing time for DNA extraction and qPCR is less compared to microscopy, with the recent development of automated extraction platforms for the isolation of DNA from environmental samples, a larger number of samples can be processed in a shorter duration.

Our study has some key limitations. First, this was a cross-sectional study with the goal of developing and validating lab and field methods for soil STH surveillance. A much larger study including longitudinal sampling across broad geographic areas is needed to validate environmental surveillance of STH as a tool for monitoring community-level infection prevalence, with sampling across rainy and dry seasons. Second, we had a higher rate of IAC failures in Kenya compared to in India and Benin, which saw almost no internal extraction control failures. This led to the removal of 29 samples (15%) from analyses of STH in soil for Kenya.

A major advantage of soil surveillance for STH is the success rate for sample collection is significantly higher compared to human stool collection. Stool sampling requires enumerators to visit participating households first to drop off a collection kit, followed by multiple return visits to successfully collect the sample. Soil sampling can be done at a single visit, concurrent with other data collection. At baseline, stool sampling among consenting participants in the DeWorm3 cluster-randomized controlled trial had an 89.8% (6092/6783) success rate in Benin and an 87.3% (6152/7054) success rate in India. In Kenya, the stool sampling success rate for this pilot study was 85%. Notably these stool sampling success rates were achieved through extensive community sensitization and visiting households up to three times for sample retrieval. In contrast, the soil sampling success rate at participating households was 95% (98/103) in India, 100% (106/106) in Benin, and 100% (120/120) in Kenya. As we observed STH prevalence in water source soil was comparable to household entrance soil in this study, sampling at water sources may be a more efficient strategy requiring fewer samples than household entrances. However, the presence of soil at the primary drinking water source was a limiting factor for soil sampling in India; soil was only present at 52% (54/103) of water sources in India, compared to 99% (105/106) in Benin and 93% (111/120) in Kenya. In India, many rural villages in the study site had concrete or cow dung covering much of the area around community water sources. An alternative sampling strategy for concrete surfaces could include sweeping a larger specified area to collect soil. Given that MDA programs are delivered at the community level, it’s likely most efficient to also assess STH prevalence through environmental sampling at public sites or common spaces that can capture STH circulating in the community rather than at an individual household.

Our study provides new evidence on the utility of measuring STH in environmental reservoirs by comparing STH prevalence between spatially and temporally matched stool and soil samples. We found that the dominant STH species responsible for human infection in each study site were also the dominant species detected in soil. Using species-specific Taqman-based qPCR assays, soil sampling can identify the presence of human-infecting STH species in order to target high-burden communities for appropriate interventions. While these formative results show promise, additional research is needed to validate soil surveillance strategies across larger populations, geographic regions, and seasons. Future work is needed to optimize the number and location of soil samples needed to predict human infection prevalence within meaningful thresholds (e.g. < 2% prevalence to indicate transmission interruption for a particular species) [23]. Soil surveillance could be a cost-saving tool for monitoring STH prevalence over time, including detecting recrudescence during and after MDA programs. National programs implementing MDA and STH surveys can leverage qPCR capabilities at regional or national reference laboratories (established for surveillance programs such as SARS CoV2 or tuberculosis) to develop more accurate estimates. Data on STH in environmental reservoirs could be useful in influencing critical programmatic decisions, such as when MDA should be renewed, reduced, or stopped altogether. Environmental surveillance of STH transmission–rather than monitoring MDA program coverage–could strengthen longitudinal and localized monitoring efforts for identifying transmission breakpoints and determining whether a sustained break in transmission has been achieved [53]. Community-level soil surveillance for STH could be a feasible, affordable, and efficient strategy for districts and programs looking to enhance their MDA program monitoring as they move beyond morbidity control and towards transmission interruption.

## Supporting information

S1 Text Appendix A. Assay Development Methods. **Appendix B.** Inhibition Testing. **Appendix C.** Internal Amplification Control Results. **Appendix D.** Sanger sequencing of *Ancyclostoma duodenale* qPCR positive soil DNA samples. **Appendix E.** DNA Extraction Protocol. **Appendix F.** qPCR Protocol. **Fig A.** Scatterplot of egg counts by light microscopy and cycle threshold (Ct) values by qPCR for soil samples. **Table A.** Sample-level soil-transmitted helminth (STH) prevalence in soil collected from household entrances and household drinking water sources by microscopy and qPCR, and STH prevalence in stool samples by qPCR. **Table B.** Agreement of ddPCR and qPCR for the detection of STH in soil among a subset of soil samples from India and Benin (DeWorm3 study sites) randomly selected for ddPCR comparison. **Table C.** Agreement of microscopy and qPCR for the detection of STH in soil, where Kappa statistics indicate agreement from poor, slight, fair, moderate, substantial, to perfect. **Table D.** Soil characteristic variables included in bivariate regressions, where outcome variables were qPCR detection (presence/absence) of soil-transmitted helminths (STH) in soil samples. **Table E.** Soil sample characteristics by sample type (soil collected from household entrance versus household water source). **Table F.** Agreement in soil-transmitted helminth detection between duplicate wells for a subset of samples randomly selected for ddPCR and qPCR. **Table G.** Sanger sequencing results from soil samples collected in India that were positive for *A*. *duodenale* based on qPCR. Raw data was analyzed using Sequencher V 5.4.6. **Table H.** Primer and probe sequences used in three-country field study for detection of soil transmitted helminths via qPCR. **Table I.** Results from primer optimization of candidate assays for *A*. *duodenale*. **Table J.** Specificity testing of *A*. *duodenale* and *A*. *ceylanicum* qPCR assays.
(DOCX)
